# Multi-platform imaging in ABCA4-Associated Disease

**DOI:** 10.1038/s41598-019-42772-z

**Published:** 2019-04-23

**Authors:** Lijuan Chen, Winston Lee, Jose Ronaldo Lima de Carvalho, Stanley Chang, Stephen H. Tsang, Rando Allikmets, Janet R. Sparrow

**Affiliations:** 1 Department of Ophthalmology, Columbia University, New York, New York United States; 2 Department of Ophthalmology, People’s hospital of Putuo District, Shanghai, China; 3 Departament of Ophthalmology, Empresa Brasileira de Servicos Hospitalares (EBSERH) - Hospital das Clinicas de Pernambuco (HCPE), Federal University of Pernambuco (UFPE), Recife, Brazil; 4 Department of Ophthalmology, Federal University of São Paulo (UNIFESP), São Paulo, Brazil; 5 Department of Pathology and Cell Biology, Columbia University, New York, New York United States

**Keywords:** Disease genetics, Retinal diseases

## Abstract

Fundus autofluorescence (FAF) imaging is crucial to the diagnosis and monitoring of recessive Stargardt disease (STGD1). In a retrospective cohort study of 34 patients, we compared FAF imaging platforms varying in field size (30° and 55°: blue/SW-AF and NIR-AF; 200°: ultrawide-field, UWF-AF), excitation wavelength (488 nm, blue/SW-AF; 532 nm, UWF-AF and 787 nm, NIR-AF) and image processing. Due to reduced absorption of 532 nm and 787 nm light by macular pigment, foveal sparing was more readily demonstrable by green/UWF-AF and NIR-AF imaging. Prominent in green/UWF-AF images is a central zone of relatively elevated AF that is continuous inferonasal with a demarcation line bordering lower AF nasally and higher AF temporally. This zone and border are more visible in STGD1 than in healthy eyes and more visible with green/UWF-AF. With the development of AF flecks, inferonasal retina is initially spared. Central atrophic areas were larger in NIR-AF images than in blue/SW-AF and green/UWF-AF images and the presence of a contiguous hyperAF ring varied with imaging modality. Flecks visible as hyperAF foci in blue/SW-AF images were also visible in green/UWF-AF but were often hypoAF in NIR-AF. Since disease in STGD1 often extends beyond the 30° and 55° fields, green/UWF-AF has advantages including for pediatric patients. The imaging platforms examined provided complementary information.

## Introduction

Recessive Stargardt disease (STGD1) is the most common inherited form of juvenile macular degeneration and is caused by pathogenic variants in the *ABCA4* gene^1^. It is characterized by progressive loss of central vision that typically occurs in the first two decades of life. Fundus autofluorescence (AF) as a non-invasive imaging technique often enables the detection of abnormalities that are invisible by standard color fundus photography. As such fundus AF is used for the diagnosis and monitoring of disease progression in STGD1.

Two sources of fundus AF are inherent to the retina. Short-wavelength blue autofluorescence (488 nm excitation; blue/SW-AF) originates from bisretinoid lipofuscin that in a healthy eye is situated within the retinal pigment epithelium (RPE)^2–4^. The second autofluorescence modality is generated with near-infrared excitation (NIR-AF; 787 nm). The maximum NIR-AF signal is observed in the center of the fundus and has a width of ~8°^5–7^. This signal is considered to be derived primarily from RPE melanin with a lesser contribution from choroidal melanocytes. This area of elevated NIR-AF corresponds to the area of high melanin optical density observed in color fundus photographs and with the area of reduced SW-AF that surrounds the area of strongest macular pigment absorption^5^. RPE cells are taller in this region and become diminished in height with further distances from the fovea^8^. Further evidence to support the assignment of this NIR-AF to melanin is provided by the NIR-AF emission associated with melanocytic choroidal nevi^5,7^, RPE melanosomes^9^, cutaneous melanin^10^ and the pigmented epithelium of the iris^5^, together with the window-defect created by a full thickness macular hole^5^. Moreover, in NIR-AF images acquired from carriers of X-linked ocular albinism (GPR143/OA1), the fundus presents as a mosaicism in which patches of NIR-AF signal correspond to pigmented areas and alternate with patches of darkness^11^. Ocular imaging of melanin with near-infrared AF (NIR-AF; 787 nm) is approximately 60 times less sensitive than blue/SW-AF autofluorescence imaging^5^. Imaging platforms that have been developed to capture fundus AF include multiple versions of the confocal scanning laser ophthalmoscope (cSLO) that capture standard 30° and 55° fields and an ultra-wide-field (UWF) ophthalmoscopic technology that can image a 200° field.

The objective of the studies described here was to elucidate novel disease features in STGD1 by using three different fundus AF imaging systems that are currently used in clinical centers. The signal originating primarily from bisretinoid lipofuscin was imaged with two excitation wavelengths: blue/SW-AF (488 nm) operating within 30° and 55° fields and green excitation (532 nm) covering an UWF (200°). The signal attributed chiefly to melanin was imaged as near-infrared autofluorescence (NIR-AF) recorded with 787 nm excitation. In the healthy eye and in patients with STGD1, blue/SW-AF or NIR-AF can provide different but complementary information with respect to the extent and location of disease-related abnormal AF. Since green/UWF-AF is also now used in the clinic and the ease of imaging with the green/UWF-AF platform (Optos) has advantages including for patients presenting with early-onset disease such as STGD1, but there are no comparisons of blue/SW-AF, NIR-AF and green/UWF-AF images in the literature.

## Results

### STGD1 patient cohort

Patients with both a clinical and genetic molecular diagnosis of STGD1 were studied retrospectively. Thirty-four patients (68 eyes) were included and for all patients blue/SW-AF, NIR-AF and green/UWF-AF images were available. All images were acquired between April 2017 and June 2018. Demographic, selected clinical, and genetic data are presented in Table 1. Patients were assigned to one of four groups based on the appearance of *en face* fundus autofluorescence images. Patient groups are described in Table 2. Patterns of autofluorescence were compared to healthy eyes.

**Table 1:** Demographic, clinical, and genetic summary of the Stargardt disease patient cohort.

Group	Patient	Gender	Age	Ethnicity	BCVA		ABCA4	ABCA4
					OD	OS	Mutation 1	Mutation 2
Group I	1	F	36	White	20/150	20/150	p.G1961E	p.P1380L
	2	M	15	White	20/25	20/25	p.G1961E	p.V615A
	3	M	10	White	20/50	20/50	p.[L541P;A1038V]	p.G1961E
	4	M	18	White	20/100	20/125	p.W1772*	p.G863A
	5	M	34	White	20/25	20/30	p.[L541P;A1038V]	
	6	F	36	White	20/30	20/20	p.G1961E	
	7	M	31	White	20/100	20/70	p.G1961E	p.Q1003*
	8	F	16	White	20/50	20/50	p.L541P	p.G1961E
	9	M	14	White	20/70	20/100	p.V989A	c.2918 + 5 G > A
Group II	10	M	51	White	20/100	20/100	p.G88R	p.N1868I
	11	M	53	Hispanic	20/20	20/50	p.G991R	c.570 + 1798A > G
	12	M	26	White	20/40	20/60	p.C54Y	c.[5196 + 1137 G > A;1555–2745 A > G]
	13	F	18	White	20/25	20/25	p.R1108C	p.Q1412*
	14	M	13	White	20/25	20/25	p.[L541P;A1038V]	p.L2027F
	15	F	68	White	20/200	20/70	p.L1014R	
	16	M	72	White	20/60	20/40	p.W663*	c.[4253 + 43 G > A;6006_609T > A]
	17	F	58	White	20/100	20/80	p.R2077W	p.N1868I
Group III	18	M	17	White	20/200	20/200	p.[L541P;A1038V]	
	19	M	59	White	20/40	20/40	p.R1705W	p.N1868I
	20	F	20	White	20/125	20/125	p.T972N	p.L2027F
	21	F	13	White	20/300	20/250	c.5312 + 1 G > A	p.R2030*
	22	F	17	White	20/150	20/150	p.P1380L	
	23	M	13	White	20/400	20/400	p.[L541P;A1038V]	
	24	F	52	White	20/150	20/100	p.G1961E	c.1100-1 G > A
	25	M	17	White	20/40	20/50	p.[L541P;A1038V]	p.L2027F
	26	M	15	White	20/200	20/200	p.W855*	p.T1526M
	27	M	63	White	20/25	20/20	p.R681*	
Group IV	28	M	19	Asian	20/400	20/400	p.R408*	c.5935delA
	29	M	23	White	CF	CF	c.4537delC	p.R107*
	30	M	55	White	20/30	20/400	p.R602Q	p.N1868I; p.C1490Y
	31	F	47	White	20/25	20/125	p.N965Y	p.P1486L
	32	M	70	White	20/40	20/40	p.R653C	p.N1868I
	33	M	35	Indian	CF	20/300	c.[859-9 T > C;66 G > A]	p.Q2220*
	34	M	54	Asian	CF	20/40	p.R24H	c.1561delG

OD, right eye; OS, left eye; BCVA, best-corrected visual acuity; F, female; M, male.

**Table 2:** Characteristics of Patient Groups.

	Group I	Group II	Group III	Group IV
N (number)	9	8	10	7
males, females	6,3	5,3	6,4	6,1
age (mean ± SD)	24.5 ± 10.07	44.87 ± 22.80	28.6 ± 20.56	43.29 ± 18.48
features of images	central area of atrophy with or without flecks extending to the vascular arcades	central atrophy and varying degrees of flecks, with inferonasal sparing	central atrophy with widespread flecks	severe disease with central atrophy and widespread dark mottling

### Fundus autofluorescence in healthy eyes

**Figure 1:**
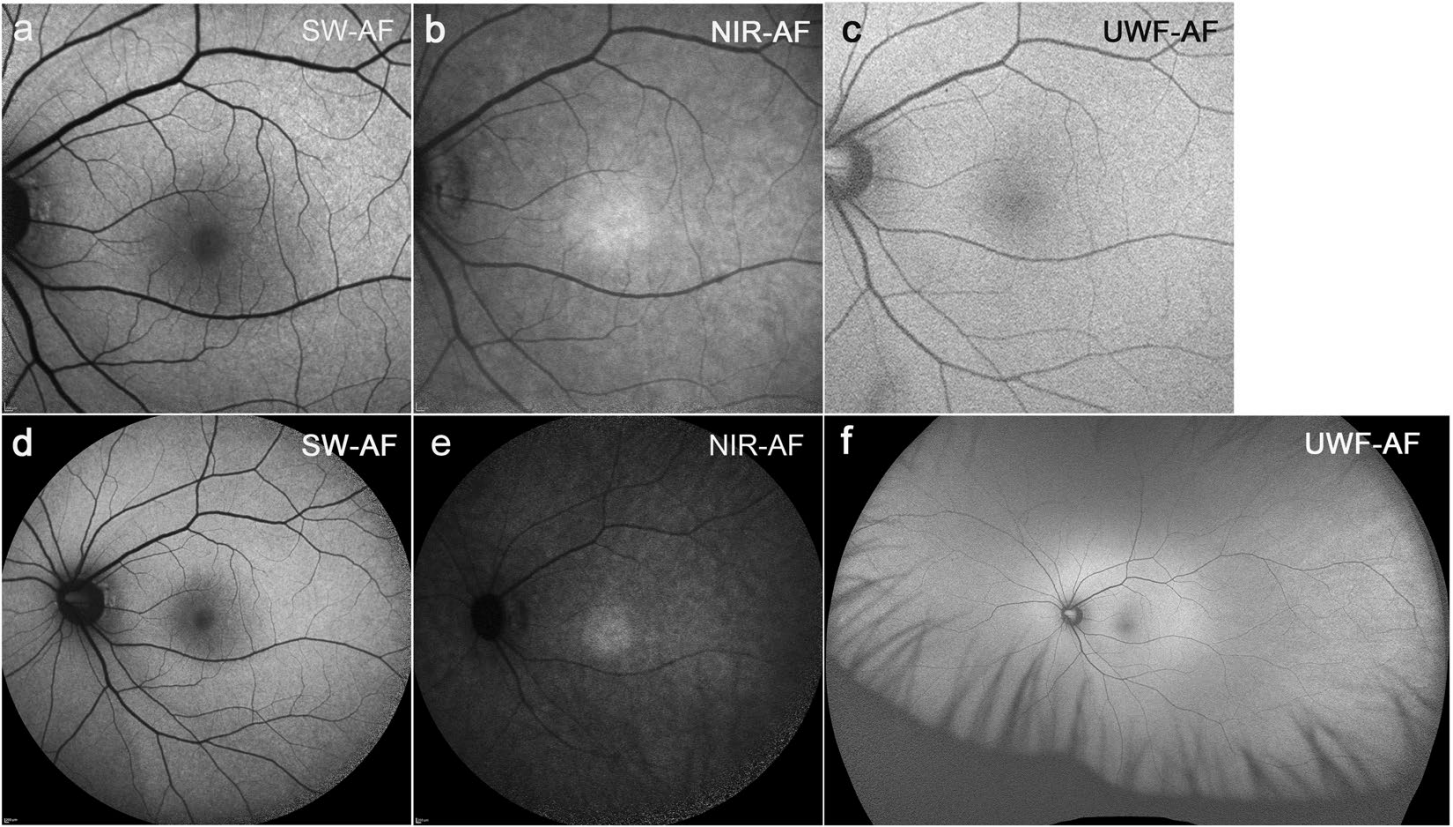
Representative fundus autofluorescence images of a healthy eye. **(a)** Blue/SW-AF; 30° × 30° field. **(b)** NIR-AF; 30° × 30° field. **(c)** Green/UWF-AF; 30° × 30° field. **(d)** Blue/SW-AF; 55° × 55° field. **(e**) NIR-AF; 55° × 55° field. **(f)** Green/UWF-AF; 200° × 200° field.

In healthy eyes (Fig. 1), blue/SW-AF (488 nm) images are distinguished by a central area of reduced AF that extends to an eccentricity of 8° due to absorption of the blue excitation light by macular pigment and melanin. Optos images recorded with green light excitation also exhibit a central area of reduced AF, but this area is smaller than in the blue/SW-AF image and not as dark. The NIR-AF image has essentially the opposite distribution of blue/SW-AF signal with elevated intensity centrally that is attributable to increased melanin optical density.

### Fundus autofluorescence in Group I patients

**Figure 2:**
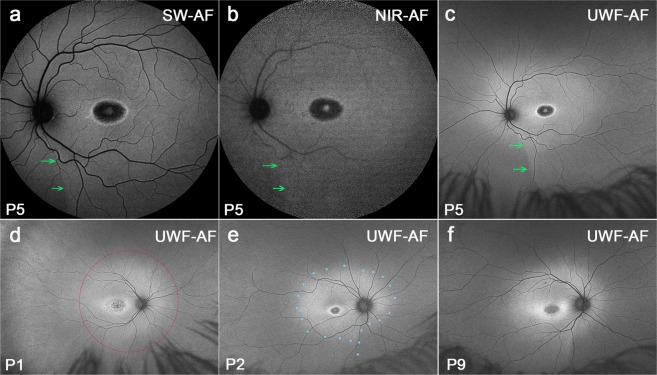
Representative images of optic fissure and oval-shaped zone of relatively higher AF. Group 1 Patients (P) 5, P1, P2, P9. The optic fissure is visible as a demarcation line (green arrows) in fundus autofluorescence. (**a**–**c**) A horizontal oval-shaped zone (blue dotted line in e) of relatively higher AF, a inferior-nasal wedge and demarcation line are most visible in green/UWF-AF images acquired with Optos. (**c-f**) A red circle indicating the 55° field was superimposed on d.

Images assigned to Group I presented with a central area of atrophy with or without flecks extending to the vascular arcades. Most patients carrying G1961E mutations were assigned to Group I on the basis of phenotype. The most noticeable feature of the green/UWF-AF images in Group I is the presence of an horizontal oval-shaped zone of relatively higher AF that envelopes the macula, vascular arcades and optic disk and is continuous inferonasally with a demarcation line that straddles areas of lower AF nasally and higher AF temporally (Fig. 2a–c; green arrows). Because of contrast with surrounding peripheral retina in the wide-field view, it was clear that the region with increased AF extended almost to the limits of a 55° field, with an outer boundary that assumed an oval shape(Fig. 2d–f).

**Figure 3:**
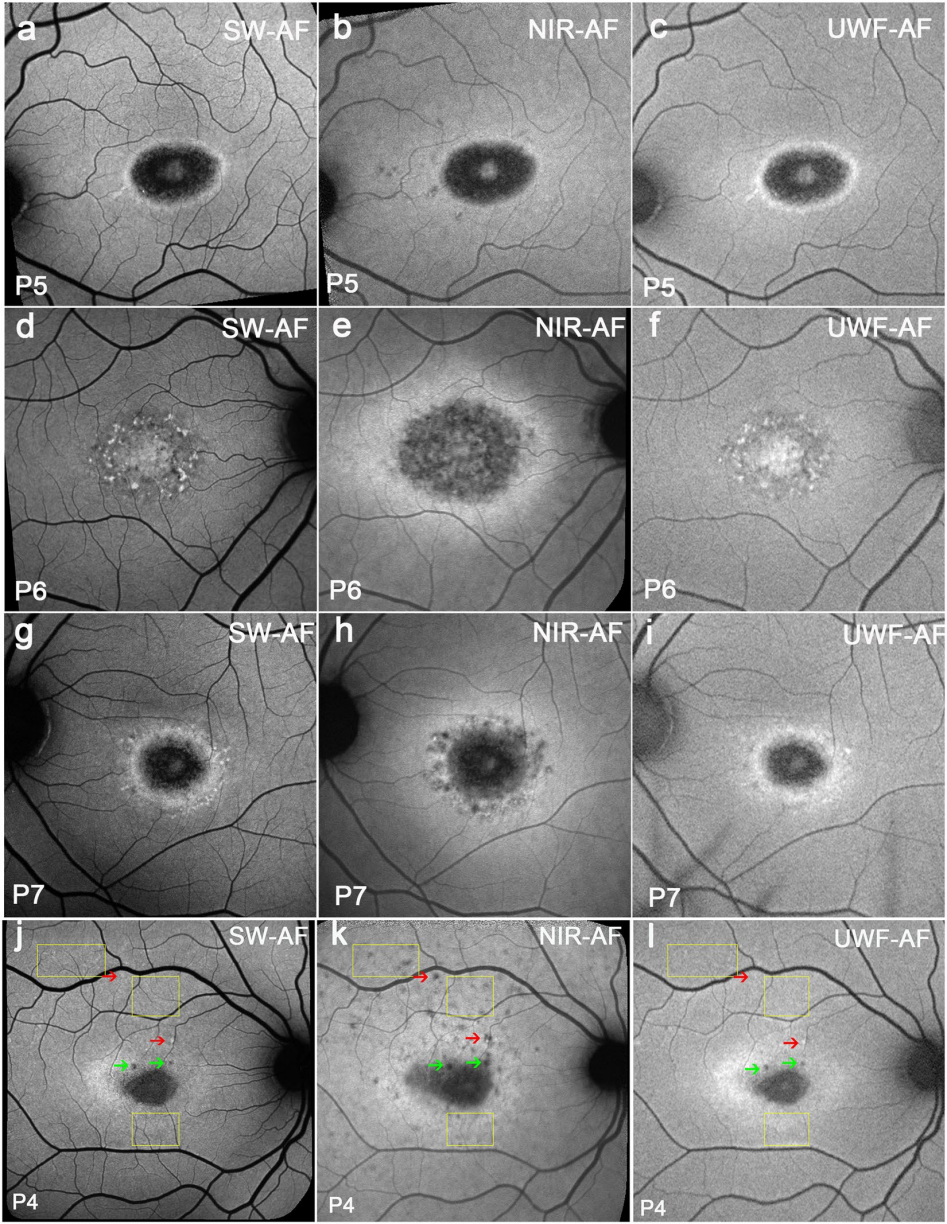
Representative images of the macula acquired with 488 nm (blue/SW-AF), 787 nm (NIR-AF) and 532 nm (green/UWF-AF) excitation. Group 1. Patients (P) 5, P6, P7, and P4. (**a**–**c**) A similar central hyperAF ring is visible in all modalities. (**d–f**) An intense hyperAF ring is only visible in the NIR-AF image. (**g**–**i**) A hyperAF ring is more visible in the NIR-AF image than in blue/SW-AF and green/UWF-AF images. (**j**–**l**) Many flecks (yellow rectangles) are barely detectable in the blue/SW-AF image, hypoAF in the NIR-AF image, and not detectable in green/UWF-AF. Other flecks (red arrows) are bright in blue/SW-AF, hypoAF in NIR-AF and bright in green/UWF-AF. Flecks (green arrows) can also be dark in all modalities.

**Figure 4:**
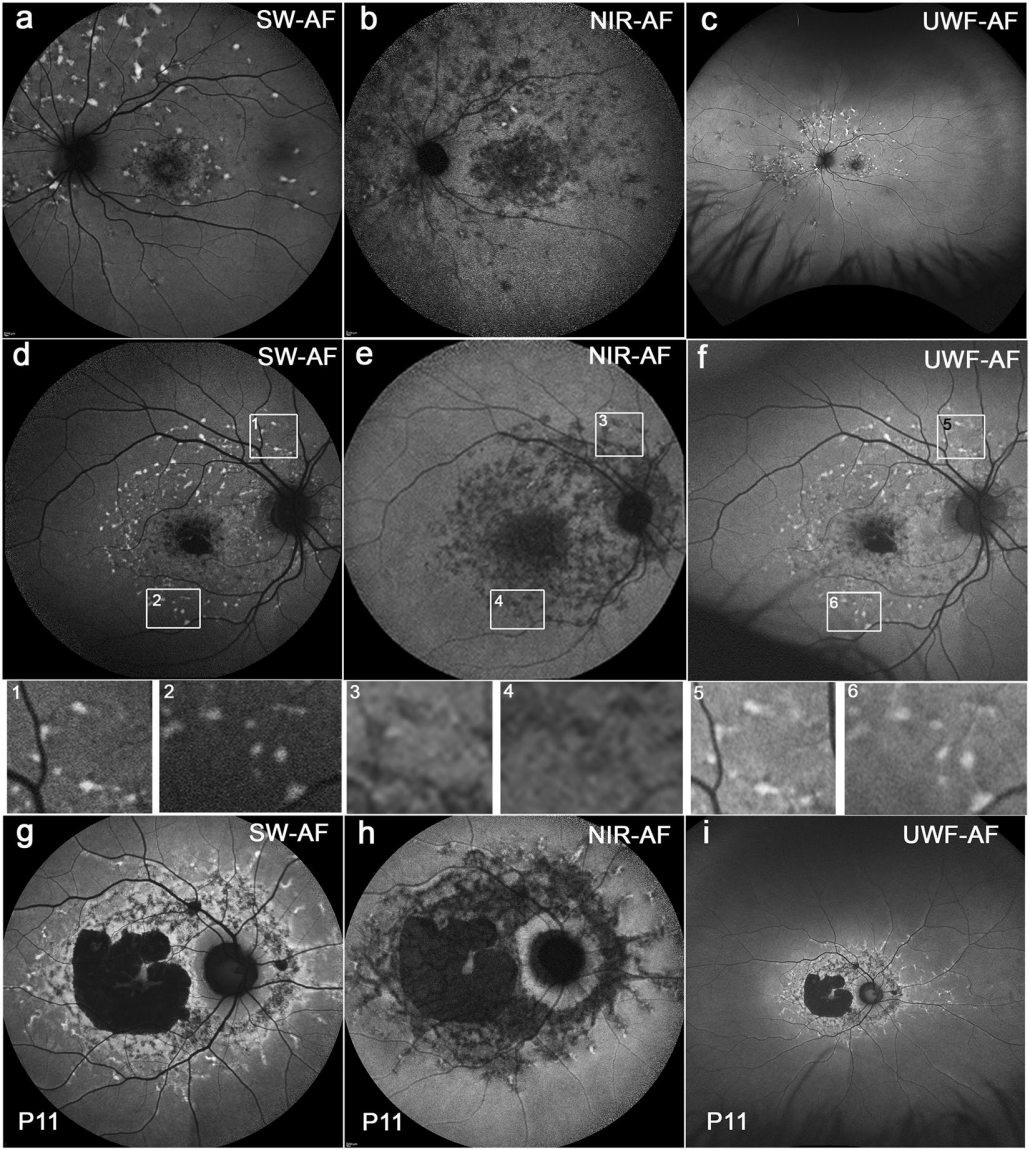
Representative images of STGD1 patients having central atrophy and varying degrees of flecks with inferior sparing. Group II. Patients (P) 17, P10 and P11. In the green/UWF-AF images, the flecks are present within the irregular horizontal ellipse-shaped fundus area exhibiting brighter AF than in the surround. Most flecks present as hyperAF foci in blue/SW-AF images (**d**) while being dark in NIR-AF images. (**e**) These flecks were also hyperAF on green/UWF-AF. (**f**) Insets below are magnifications of areas shown in d, e, f (rectangles). The borders of the foci can be blurred (inset 5). Delicate bright lines appear to link flecks (inset 6), consistent with the dark lines in the NIR-AF images. Dash-like flecks are hyper-AF in blue/SW-AF (**g**). Flecks are dark and contiguous with central areas of atrophy in NIR-AF images (**h**). The branching flecks often extended into the periphery, being distributed along the blood vessels in green/UWF-AF images (**i**). Foveal sparing and sparing of the peripapillary region was observed (**g**–**i**).

All the patients showed a central hypoAF area that in some patients appeared to be larger in NIR-AF images (Fig. 3e,h) than in blue/SW-AF (Fig. 3d,g) and green/UWF-AF images (Fig. 3f,i). This central hypoAF area was surrounded by a hyperAF ring in NIR-AF images. In 4/9 patients a similar hyperAF ring was visible in all three modalities (Fig. 3a–c); 1/9 patients had an intense hyperAF ring in NIR-AF images (Fig. 3e), while the ring was not visible in blue/SW-AF (Fig. 3d) and green/UWF-AF images (Fig. 3f) and 4/9 patients had more intense hyperAF in NIR-AF (Fig. 3h) than in blue/SW-AF (Fig. 3g) and green/UWF-AF images (Fig. 3i). In group I patients, some had central atrophy with flecks (4/9). Flecks that were hypoAF in NIR-AF, could be either hyperAF in blue/SW-AF (Fig. 3j, red arrows) and UWF-AF images (Fig. 3l, red arrows) or hypoautofluorescent in blue/SW-AF (Fig. 3j, green arrows) and UWF-AF images (Fig. 3l, green arrows).

### Fundus autofluorescence in Group II patients

Images of Group II presented with a central area of atrophy, with varying degrees of flecks and inferonasal sparing (Fig. 4). Flecks in blue/SW-AF and NIR-AF images extended radially from the fovea; more flecks were detectable in the superior (above the vascular arcades) and temporal macula while areas inferonasal to the optic disc were spared. In green/UWF-AF images these flecks were located within the horizontal-ellipse shaped area of bright fundus AF (8/8 patients). The demarcation line extending inferior to the optic disc was visible in the NIR-AF images (P17) in addition to being visible in blue/SW-AF and green/UWF-AF images. Disease-associated AF patterns appeared to respect this boundary (Fig. 4a–f).

Most flecks presented as hyperAF foci with distinct boundaries in blue/SW-AF images while being dark in NIR-AF images. These flecks were also hyperAF on green/UWF-AF (Fig. 4f), but the borders of the foci could be blurred and hyperAF lines appeared to connect adjacent foci, consistent with the dark lines on NIR-AF (Fig. 4e). Additionally, one patient (P11) exhibited central atrophy that was contiguous with dark flecks in NIR-AF images (Fig. 4h). Foveal sparing and sparing of the peripapillary region was observed in all 3 imaging platforms (Fig. 4g–i). although it was more evident in NIR-AF images (Fig. 4h).

### Fundus autofluorescence in Group III patients

**Figure 5:**
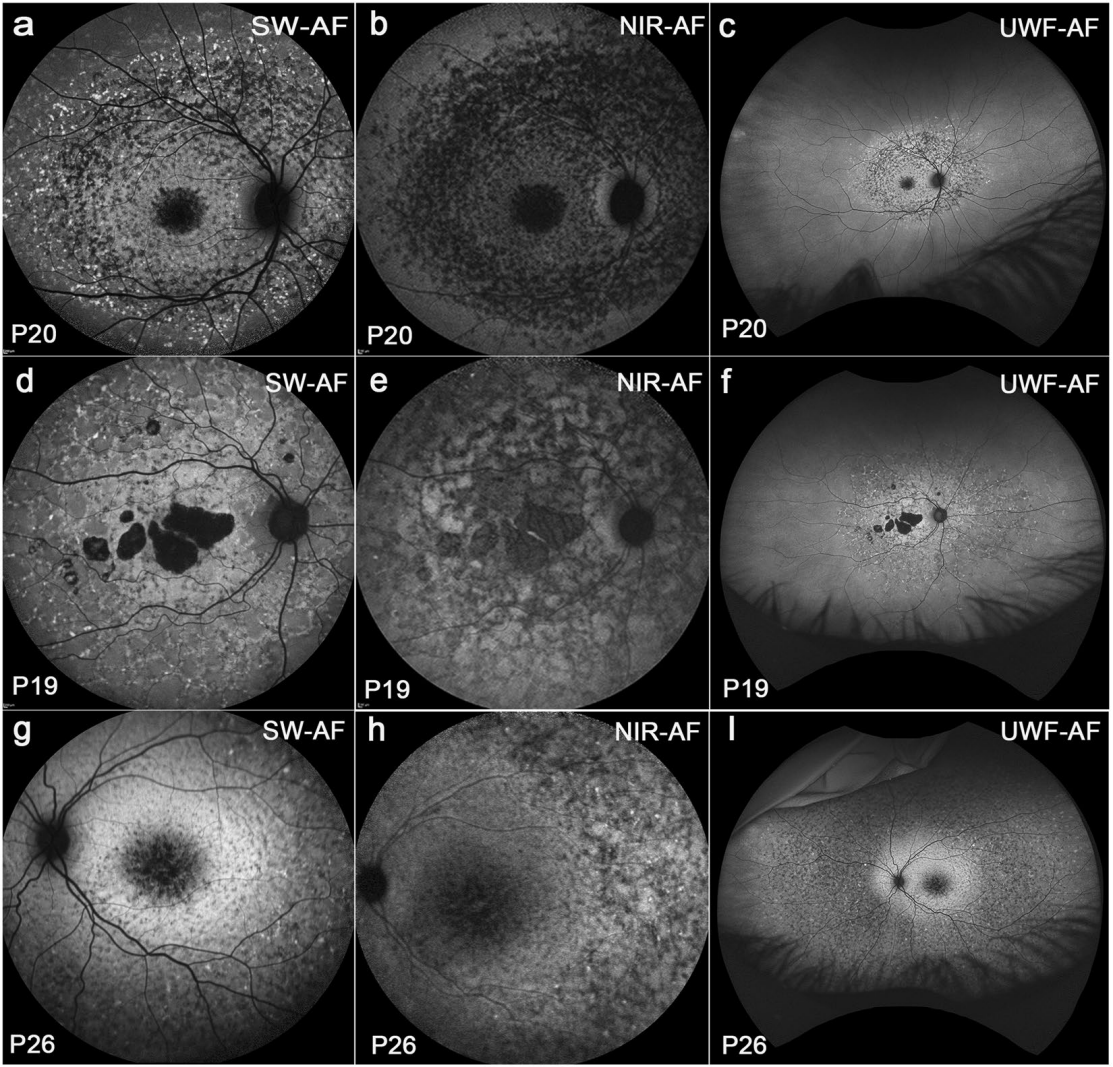
Widespread flecks with different shapes and distributions. Group III. Patient (P) 20, P19 and P26. Blue/SW-AF(**a**,**d**,**g**). (**a**) Flecks in the macula are round and dark. Peripheral to the macula flecks are hyperAF and circularly arranged. (**d**) Flecks can be contiguous and hyperAF. (**g**) Dense small flecks were hypoAF with a few hyperAF spots scattered among and a visible hyperAF ring noted. NIR-AF (**b**,**e**,**h**). (**b**) Flecks are typically hypoAF. (**e**) Flecks are dark and contiguouas with central areas of atrophy. (**h**)The hyperAF ring in g was not detected. Green/UWF-AF (**c**,**f**,**i**). (**c**) Peripheral flecks were readily visible. (**f**) Branching flecks often extended into the periphery. (**i**) An irregular oval-shaped area of increased autofluorescence is present in green/UWF-AF image.

Images of Group III exhibited central atrophy with widespread flecks (Fig. 5). In this group, flecks had different shapes and fleck distribution varied; the configurations of atrophy also varied. Flecks could be either round or elongated. In blue/SW-AF images an oval area of central macular atrophy could be surrounded by dark flecks with hyperAF flecks being outermost and extending circularly and peripheral to all four quadrants. The latter flecks were typically hypoAF in NIR-AF (Fig. 5b,e,h). In green/UWF-AF (Fig. 5c) peripheral flecks were readily visible and, at an eccentricity greater than 55° could be seen against an oval-shaped brighter fundus region. Some dash-like flecks were hyper-AF in blue/SW-AF images and often being contiguous, they created a branching or net-like appearance. These fleck profiles in NIR-AF images (Fig. 5e) were dark and they were usually contiguous with central areas of atrophy. In green/UWF-AF images (Fig. 5f) it was apparent that branching flecks often extended out into the periphery distributing along the blood vessels. Some patients had dense small flecks extending to the far periphery; these flecks were typically hypoAF in blue/SW-AF (Fig. 5g) and green/UWF-AF (Fig. 5i) images with a few hyperAF spots scattered among them in blue/SW-AF and green/UWF-AF images. The hyperAF ring visible in blue/SW-AF images (Fig. 5g) of P26 could not be detected in NIR-AF images (Fig. 5h) but in green/UWF-AF (Fig. 5i) images was present as an irregular oval-shaped area of high autofluorescence. In blue/SW-AF and NIR-AF images, only those flecks within the 55° field could be seen but in green/UWF-AF images, it was obvious that flecks could form well beyond this range (10/10 patients).

Six (6/10) patients were younger than 17 years in this group. One patient (P18) had disease onset at 10 years of age. Blue/SW-AF at baseline showed a central area of atrophy surrounded by a hyperAF ring, with some flecks in the superonasal field (Fig. S1a). After a 3 year-interval, flecks had spread well beyond the 30° field and were visible in the 55° field (Fig. S1b). After an additional 3 years, the flecks were more widespread than was visible within the 55° field photographed with the Spectralis (Fig. S1c). Inspection of the green/UWF images revealed that the disease extended into far peripheral retina (Fig. S1d).

### Fundus autofluorescence in Group IV patients

**Figure 6:**
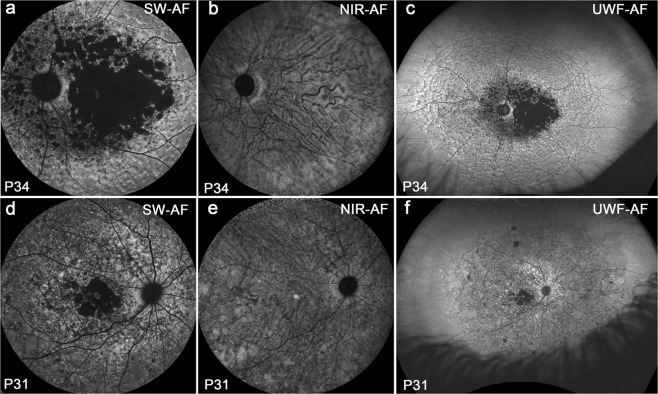
Severe disease with central atrophy and widespread dark mottling. Group IV. The border of atrophy is clearly visible in SW-AF (**a**,**d**); it is not visible in NIR-AF (**b**,**e**). Choroidal blood vessels are distinctly visible in the area of atrophy in NIR-AF (**b**,**e**). Disease is more widespread than is visible within 55° field and has a reflectance-like appearance in Green/UWF-AF (**c**,**f**). Foveal sparing is visible in all three modalities.

Images of Group IV revealed severe disease with central atrophy and widespread dark mottling (Fig. 6). In four patients with severe atrophy, the border of the atrophic area was clearly visible in both blue/SW-AF (Fig. 6a,d) and green/UWF-AF (Fig. 6c,f). Although good contrast between atrophic versus non-atrophic areas is usually obtainable with NIR-AF imaging^12^, this boundary was not visible in NIR-AF images (Fig. 6b,e) of 2 patients in this group. One reason for this could be that in the presence of severe RPE atrophy, the contribution of choroidal melanin to the NIR-AF signal was high relative to the total NIR-AF signal in these patients^5^. Inspection of the green/UWF images revealed that the disease was more widespread than is visible within the 55° field photographed with the Spectralis. The size of the area of hypoautofluorescence indicative of atrophy was much larger in NIR-AF images when compared to blue/SW-AF and green/UWF-AF.

## Discussion

The findings in this study utilize lipofuscin and melanin as two major sources of fundus fluorophores that can be harnessed to track disease progression in STGD1. In the healthy eye, lipofuscin is kept to a minimum in photoreceptor cells due to a fully functional ABCA4 protein, efficient reduction of retinaldehyde, daily shedding of outer segment membrane, and phagocytic transfer to RPE^2,13^. Melanin is a protective agent in the retina as it reduces light scatter and light-mediated toxicity^14–16^.

Green/UWF-AF imaging with the Optos revealed that disease processes are often more wide-spread than is visible in a 30° × 30° field in STGD1. We found that 9/34 patients (26%) had lesions confined to the macula; 7/34 patients (21%) had lesions outside the macula but limited to the 55° field and 18/34 (53%) had lesions extending outside the 55° field. Some of these peripheral lesions can present as dark atrophy with a distinct border and can be scattered like satellites in the far periphery. This is consistent with other reports^17–19^ indicating that the majority of patients with STGD1 have changes in the peripheral retina. Thus wide field examination should to be integrated into the management of SDGT1.

Retinal flecks are a prominent clinical feature of STGD1. Consistent with our previous research^13,20^, we noted that in NIR-AF images (Fig. 3k) flecks were often hypoAF before any indication of hyperAF in blue/SW-AF images (Fig. 3j). Flecks that were bright in blue/SW-AF were also bright in green/UWF-AF and flecks patterns were similar. Moreover, in blue/SW-AF and green/UWF-AF images, some flecks first appeared to be hyperAF and then become hypoAF as disease progressed (Fig. S1). This change in fluorescence likely represents bleaching of the underlying fluorophore. Taken together we interpret these observations as evidence that at positions of flecks, the melanin signal (NIR-AF) of RPE is reduced or absent due to atrophy of these cells. Consistent with earlier studies we also observed that flecks often expand centrifugally outward from the macula^21–23^. It has been suggested that details in an image are more difficult to resolve in images acquired with the Optos instrument, but we found that flecks visible in blue/SW-AF images were also detectable in Optos images.

The most pronounced difference between the blue/SW-AF and green/UWF images is the relative brightness of the fovea in green/UWF images due to reduced absorbance by macular pigment. The carotenoids that constitute macular pigment have a combined absorption maximum of ~460 nm^24,25^. At the excitation of 532 nm utilized by the Optos, macular pigment absorbance is significantly reduced. The spatial distribution of macular pigment is visible in the blue/SW-AF images of the fundus as a dark spot in the fovea that is about 2–3° in diameter. Macular pigment density then decreases to become optically undetectable at 6° to 8° eccentricity^26,27^. Autofluorescence in the blue/SW-AF and green/UWF images is also attenuated by RPE melanin but this effect is more pronounced with the 488 nm excitation (blue/SW-AF). This area of higher melanin optical density presents as the zone of brightness in NIR-AF (787 nm) images (Fig. 1) that extends within an area 8° in diameter and corresponds to the area of higher melanin optical density that is observed in color and blue/SW-AF images^5^. Due to the reduced absorption by macular pigment, the longer excitation wavelength (532 nm) used for Optos-acquired images can make preserved foveal islands more clearly visible (Figs 2c,e, 3l, 4I, 5f and 6c,f). This can also be the case for NIR-AF imaging (Figs 2b and 4b,f,h).

The distinctness of a central oval-shaped area with comparatively elevated hyperAF is an interesting feature of the green/UWF-AF images in both healthy and diseased fundus. Based on previous analysis by quantitative fundus autofluorescence (qAF), it is clear that the AF intensity in this area is further elevated in STGD1 patients as compared to healthy eyes^28,29^. In images exported from the Optos, the oval-shaped area appears particularly elongated along the horizontal axis probably because of optical distortions inherent to the green/UWF platform. This horizontally oriented oval region of AF is interrupted inferonasally by a quasi-vertical demarcation line and wedge-shaped zone of reduced AF. In 30° × 30° blue/SW-AF images Duncker *et al*., previously demonstrated the presence of this same boundary and wedge in healthy eyes^28,30^. AF levels on the nasal side were ~25% lower than temporally (at a position 20° inferior to the disc center). The authors assigned this boundary to the embryonic optic fissure. Moreover, the contrast between the temporal higher AF and nasal lower AF intensity was accentuated in STGD1 patients and unlike in healthy eyes, this demarcation line was also visible in NIR-AF images^17,28^. In the green-UWF images generated by the Optos it is clear that this demarcation line is a component of the oval-shaped area of relatively enhanced AF. Significantly, disease-related AF aberrations, such as flecks, respected this demarcation line as a boundary. Thus flecks are initially confined to the central oval-shaped area of relatively higher AF while sparing inferonasal fundus.

In this study, good FAF imaging quality was observed with blue/SW-AF, green/UWF-AF, and NIR excitation wavelengths. Nevertheless, we recognize differences between the blue/SW-AF and green/UWF-AF. As compared to the blue-AF recorded with the Spectralis, the green-AF images acquired with the Optos can have reduced uniformity, particularly in superior and inferior fundus. The latter is most often due to the acquisition of images through an undilated pupil. The Heidelberg Spectralis uses real-time averaging to reduce background noise and improve image contrast. The Optos, on the other hand, does not average images^31^. Image processing by the Optos also does not include histogram stretching, an adjustment that is available with the Spectralis and that improves contrast by remapping image intensity values to the full range (0–255) available in a greyscale image. Some distortion of Optos exported images is inevitable due to the two-dimensional representation of the three-dimensional fundus surface. Techniques to address the effect of these distortions have been published^32,33^. And finally, the darkness of the optic disc in blue/SW-AF images is explained as being due to an absence of AF molecules. However, in green/UWF-532 images the optic disc can appear bright (Fig. 6).

Nonetheless, imaging with the Optos has advantages. The use of the green excitation wavelengths decreases absorption by macular pigment thereby allowing better detection of perifoveal retina^34^. Disease onset in STGD1 is often in childhood and with early-onset can be associated with rapid spread of flecks and severe vision loss (Fig. 6). The Optos enables pathology to be viewed in a child without anesthesia and pupillary dilation and in the outpatient setting^35^. Rapid image capture is also an advantage in children^36^. Across all ages, the Optos produces a 200° view of the retina (about 82% of the surface area) that includes far peripheral retina particularly in temporal and nasal retina and without the need for montages from standard 30° images or the use of contact lenses^34^. The green light excitation (532 nm) used by the Optos is more comfortable for the patients than the bright 488 nm excitation of the Spectralis. On the other hand it should be noted that retinal light exposures employed with the Spectralis are below the maximum permissible levels recommended by the American National Standards Institute for 8 hour exposure^37,38^.

Our study has some limitations that should be considered. The numbers of patients in the cohort was relatively small and the images were evaluated by unmasked investigators (L.C., J.R.S.). In addition, the progression of disease in individual patients was not studied. A final limitation is that we did not have available to us the newest ultra-widefield imaging device of Optos (Optos *California*) which may present with higher quality images than the Daytona.

All three wavelengths are valuable for noninvasive detection of disease-associated changes in patients with STGD1. We found that important features such as foveal sparing were visible with all the modalities we studied. Every modality has its advantages and shortcomings. The blue-light excitation beam may cause patient discomfort. NIR-AF imaging provides a means to visualize RPE changes that precede blue/SW-AF abnormalities and presents high contrast, non-distorted images. Since NIR light is invisible to the subject, patient cooperation is facilitated. The Optos system has the ability to acquire images through a native non-dilated pupil with a brief image acquisition time (250 ms), which has advantages including for the monitoring of pediatric patients. We conclude that the information obtained with each of the three platforms differs in certain aspects and the combined application of three imaging systems can provide further insights into pathophysiological processes in STGD1.

## Methods

### Study population

Patients with both a clinical and genetic molecular diagnosis of STGD1 were studied retrospectively. Ages ranged from 10 to 72 years, with a mean age of 34.02 years. All patients had a complete ophthalmic examination, including best-corrected visual acuity with subjective refraction. The clinical diagnosis of STGD1 was confirmed by a retina specialist based on typical fundus features and family history. Eyes were excluded from the study if there was evidence of significant ocular media opacities, refractive errors greater than 66 diopter (D) sphere or 62 D cylinder, elevated IOP > 21 mm Hg, or a history or diagnosis of any other significant ocular comorbidities. Disease-causing variants in ABCA4 were detected by direct sequencing of the ABCA4 locus as previously described^39,40^.

All patients provided written informed consent under a protocol approved by the Institutional Review Board of Columbia University. The informed consent was obtained from all subjects or, if subjects are under 18, from a parent and/or legal guardian. The study adhered to tenets set out in the Declaration of Helsinki.

### Imaging

Pupils were dilated with topical 1% tropicamide and 2.5% phenylephrine. Blue/SW-AF images were acquired using Spectralis HRA + OCT (Heidelberg Engineering, Heidelberg, Germany) (488 nm excitation, 500 nm barrier filter) after a 20-second bleach of photopigment in AF mode. The NIR-AF (787 nm excitation, >830 nm emission) images were acquired with the Heidelberg Retina Angiograph 2 scanning laser ophthalmoscope (HRA2-cSLO, Heidelberg Engineering) using the indocyanine-green angiography mode (without injection of dye) after focus adjustment in the infrared reflectance mode. To obtain high-quality images, the eye-tracking function was utilized, and 100 single frames were averaged and saved in normalized mode. Green/UWF-AF (532 nm excitation; 200° field) images was obtained using Optos 200 Tx (Optomap Daytona, United Kingdom) in AF mode.

For analysis, all images were registered and aligned using i2k Retina software (Dual Align LLC, Clifton Park, NY, USA). For illustrative purposes, some nonaligned images were used in figures.

## Supplementary information

Multi-platform imaging in ABCA4-Associated Disease

## Data Availability

The datasets generated during and/or analyzed during the current study are available from the corresponding author on reasonable request.
