# Supplementary material for: Multi-platform imaging in ABCA4-Associated Disease
Source: Sci Rep. 2019 Apr 23;9:6436. doi: 10.1038/s41598-019-42772-z (PMC6478712; doi:10.1038/s41598-019-42772-z)
Supplement: Supplementary file 1 — Multi-platform imaging in ABCA4-Associated Disease [file 41598_2019_42772_MOESM1_ESM.pdf]

Multi-platform imaging in ABCA4-Associated Disease

Authors: Lijuan Chen[1,2], Winston Lee[1], Jose Ronaldo Lima de Carvalho-Jr[1,3,4], Stanley Chang[1] , Stephen H. Tsang[1,5], Rando Allikmets[1,5], Janet R. Sparrow [1,5]*
Affiliations:
[1]Department of Ophthalmology, Columbia University, New York, New York, United States
[2]Department of Ophthalmology, People’s hospital of Putuo District, Shanghai, China
[3]Departament of Ophthalmology, Empresa Brasileira de Servicos Hospitalares (EBSERH) - Hospital das Clinicas de Pernambuco (HCPE), Federal University of Pernambuco (UFPE), Recife, Brazil.
[4]Department of Ophthalmology, Federal University of São Paulo (UNIFESP), São Paulo, Brazil
[5]Department of Pathology and Cell Biology, Columbia University, New York, New York, United States
*Corresponding author: jrs88@cumc.columbia.edu

Supplementary Materials

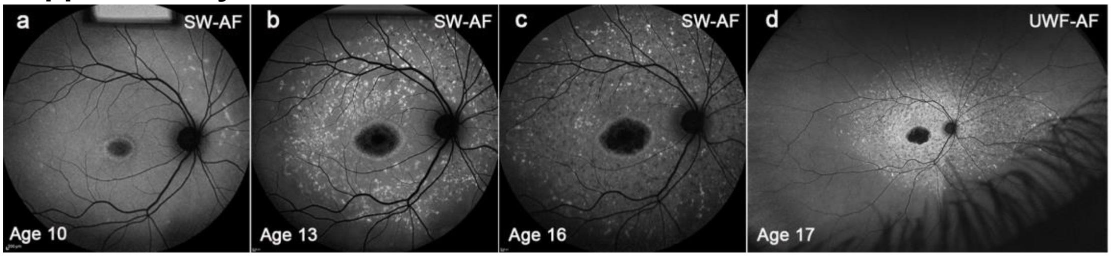

Figure S1. Longitudinal disease changes in STGD1 patient with early onset. Group III. Patient (P) 20. (a) Blue/SW-AF. At baseline, age 10, some flecks are visible in the superonasal field. (b) Blue/SW-AF. Age 13, flecks have spread throughout the 55° field. (c) Blue/SW-AF. Age 16. Flecks are more widespread than is visible within the 55° field (d) Green/UWF-AF. Age 17. Flecks extend into far peripheral retina.
